# Shaping of Hepatic Ischemia/Reperfusion Events: The Crucial Role of Mitochondria

**DOI:** 10.3390/cells11040688

**Published:** 2022-02-16

**Authors:** João S. Teodoro, Rui T. Da Silva, Ivo F. Machado, Arnau Panisello-Roselló, Joan Roselló-Catafau, Anabela P. Rolo, Carlos M. Palmeira

**Affiliations:** 1 MitoLab, Department of Life Sciences, University of Coimbra, 3000 Coimbra, Portugal; jteodoro@ci.uc.pt (J.S.T.); anpiro@ci.uc.pt (A.P.R.); 2 MitoLab, Mitochondria, Metabolism and Disease Group, Center for Neurosciences and Cell Biology, Faculdade de Medicina, University of Coimbra, 3000 Coimbra, Portugal; ruisilva_39@hotmail.com (R.T.D.S.); imachado@cnc.uc.pt (I.F.M.); 3 IIIUC–Institute of Interdisciplinary Research, University of Coimbra, Pólo II da Universidade de Coimbra, 3000 Coimbra, Portugal; 4 Experimental Pathology Department, Institute of Biomedical Research of Barcelona (IIBB), CSIC-IDIBAPS, 08036 Barcelona, Spain; arnau.panisello@iibb.csic.es (A.P.-R.); joan.rosello@iibb.csic.es (J.R.-C.)

**Keywords:** mitochondria, liver, ischemia/reperfusion, liver surgery, conditioning

## Abstract

Hepatic ischemia reperfusion injury (HIRI) is a major hurdle in many clinical scenarios, including liver resection and transplantation. Various studies and countless surgical events have led to the observation of a strong correlation between HIRI induced by liver transplantation and early allograft-dysfunction development. The detrimental impact of HIRI has driven the pursuit of new ways to alleviate its adverse effects. At the core of HIRI lies mitochondrial dysfunction. Various studies, from both animal models and in clinical settings, have clearly shown that mitochondrial function is severely hampered by HIRI and that its preservation or restoration is a key indicator of successful organ recovery. Several strategies have been thus implemented throughout the years, targeting mitochondrial function. This work briefly discusses some the most utilized approaches, ranging from surgical practices to pharmacological interventions and highlights how novel strategies can be investigated and implemented by intricately discussing the way mitochondrial function is affected by HIRI.

## 1. Introduction

### 1.1. The Liver

The liver is an organ with dozens of functions in the body, ranging from the better-known bile production to assist digestion, to others that are referenced less often, such as its involvement in carbohydrate (production and storage of glycogen; release of glucose to circulation; gluconeogenesis to generate glucose from amino acids, lactate or glycerol), protein (most circulating proteins are produced by the liver, as are most amino acids and some hormones such as angiotensinogen; hormones and other circulating proteins are broken down in the liver) or lipid (production of cholesterol, lipogenesis and triglyceride synthesis) metabolism. Other functions involve the detoxification of xenobiotics and some heavy metals, serving as a blood reservoir, producing lymph, storing vitamins and metallic ions and promoting blood immune activity (by the liver native Kupffer cells, as well as a panoply of other immune cells) [1,2].

Given of its range of roles, it is unsurprising that liver transplantation is one of the most commonly performed organ transplants. Liver injury and failure is an increasingly common event [3] and, despite improvements in survivability and transplant success, there is still a tremendous unbalance between liver donations and necessities. This is further aggravated by the fact that many (if not most) potential donors are barred from providing organs due to innate conditions, such as steatosis or steatohepatitis, cirrhosis, or cancer, just to name a few [4]. As such, novel therapeutic approaches and interventions that can increase both the pool of available donors as well as the restoration of homeostasis upon liver insult, both of which are urgently needed. One of the most common types of hepatic injury is that caused by the cut and eventual restoration of circulation, which is commonly known as ischemia and reperfusion.

### 1.2. Ischemia/Reperfusion

Hepatic ischemia-reperfusion injury (HIRI) is an accumulation of processes and events that revolve around cellular and organelle damage upon blood-flow restriction (ischemia), which is followed by a seemingly contradictory augmentation of injury upon the restoration of blood flow (reperfusion) [5]. HIRI is the main reason for complications and even mortality in the setting of hepatic surgery or transplantation [6,7]. Interestingly, despite intense investigation on the matter, the exact causes of HIRI are still unclear [8].

There are two main types of HIRI, depending on the setting of the organ at the time of ischemia, i.e., whether it is still inside the organism (warm) or outside, as is the case of transplantation (cold) [8,9]. Although in different induction settings, the pathophysiological events that take place are quite similar between the two types of HIRI [9]. While warm (normothermic, 22–25 °C) HIRI begins with hepatocyte injury and during different events such as trauma, surgery or other events that restrict blood flow, cold (ice temperature, i.e., 0–1 °C) HIRI only applies to a transplant setting, where the liver is harvested and transported in quasi-freezing temperatures in order to reduce tissue degradation. Regardless, both events result in an immune response triggered not by pathogens per se, but due to the release of pro-inflammatory signals [10], in what is known as sterile inflammation.

There are many processes involved in HIRI events, namely the metabolic shift towards an anaerobic metabolism, mitochondrial dysfunction and oxidative stress (i.e., overproduction of reactive oxygen species that overbear natural antioxidant defenses such as scavenger molecules and, more relevantly, antioxidative enzymes, which contributes to alterations in redox signaling and molecular injury [11]), calcium overload and the immune response, heavily mediated by Kupffer cell and other immune cell types’ activation, such as infiltrating neutrophils and macrophages [12,13]. As such, given the central role of the liver in metabolic and energetic whole-body homeostasis, it comes as no surprise that HIRI causes a broad-range effect on the body. In fact, during the ischemic period, the lack of oxygen shifts the ATP-generating processes from aerobic respiration (i.e., oxidative phosphorylation, OXPHOS) towards anaerobic respiration, or glycolysis. However, given enough time, the lack of oxygen results in a shutdown of redox processes, severe reduction in ATP generation capacity, and parallel acidification of the cellular milieu, given the accumulation of lactic acid and ketone bodies, resulting in what is known as metabolic acidosis. Thus, the lower pH results in enzyme, organelle and even cellular injury [14]. As expected, once blood flow is restored (reperfusion), acidification is neutralized, which is paradoxically responsible for further injury due to the activation of pH-dependent proteases and phospholipases [15,16]. In parallel, the low O_2_ pressure can also result in the elevation of cyclic AMP (cAMP) levels, resulting in the activation of cAMP-sensitive enzymes, with concomitant phosphorylation and perturbation of the function of key enzymes of the carbohydrate metabolism [17], which further contributes to the accumulation of acidic metabolites [8].

### 1.3. Mitochondrial Function and HIRI

Mitochondria are the essential players in the metabolism of eukaryotes. Virtually all of the ATP requirements of the nucleated cell derive from the processes taking place within mitochondria, i.e., the citric acid (Krebs) cycle and OXPHOS. While the Krebs cycle takes place exclusively on the mitochondrial matrix, taking in Acetyl-CoA to generate reducing equivalents (NADH and FADH_2_), OXPHOS is a process that takes place mostly within the inner mitochondrial matrix (apart from the cytochrome *c* electron transport in the intermembrane space), where electrons provided by the aforementioned reducing equivalents are transported in energetically favorable leaps along the respiratory chain (from Complexes I and II towards III and then IV) towards a molecule of oxygen, generating water. In tandem with the energetically favorable electronic transport, there is vectorial, the energetically unfavorable (against the concentration gradient) transport of protons from the matrix towards the intermembrane space, thus traversing the mostly proton-impermeable inner membrane. This generates an electrochemical gradient across this membrane (electrical due to the charge disparity, since protons are charged; chemical since protons are what make a solution acid), which is the storage of a tremendous potential energy. This energy is used to create a covalent bond between adenine diphosphate (ADP) and a molecule of ionic phosphate (Pi), generating adenine triphosphate (ATP), the cell’s energetic currency. This phosphorylation of ADP takes place at the level of the ATP synthase or complex V, a protonic channel bound to a catalytic head [18].

However, given that OXPHOS heavily relies on electron transport in a biological setting, it is thus expected that some instability is present. In fact, most of the typical cell’s reactive oxygen species (ROS) are produced in mitochondria simply as by-products of cellular respiration [19]. First considered as unwanted by-products of mitochondrial ATP generation, ROS are now ubiquitously understood as necessary, given their production does not exceed manageable levels, since mild oxidative stress is a fundamental modulator of redox signaling and the maintenance of adequate defenses and function [20]. However, it is true that excessive, prolonged ROS generation invariably results in oxidative stress, causing impaired mitochondrial function and exacerbated ROS generation, creating a snowball effect of oxidative stress that ultimately might lead to the cell’s death. Mitochondria are particularly susceptible to oxidative stress, since several of its components are severely damaged by ROS, such as mitochondrial DNA (mtDNA), which are phospholipids from the membranes or proteic elements of various metabolic pathways. For example, thiol groups within Complex I of the respiratory chain are readily oxidized, resulting in elevated ROS generation due to the mishandling of electron transport [21]. Furthermore, cardiolipin, a hallmark phospholipid of the inner mitochondrial membrane and the element most responsible for this membrane’s protonic impermeability (and, as such, for the membrane potential), is composed of highly unsaturated fatty acids, which are also prime targets for oxidation [22]. Furthermore, cardiolipin also has a regulatory role in the function of various enzymes, such as creatine kinase [23]. Metabolism is also obviously affected, and ß-oxidation is particularly susceptible, since increased acylation of proteins due to elevated matrix accumulation of acyl-CoA was found in ischemia/reperfusion events [24,25]. Another metabolic consequence is the depletion of NAD^+^, an essential co-factor for numerous metabolic pathways (such as glycolysis and Krebs cycle, just to name a few) and important enzymes such as NAD^+^-dependent sirtuins, deacetylases involved in cellular survival. Since OXPHOS is the major syphon for NADH, refreshing the NAD^+^ pool, the drastic reduction in NADH consumption helps in this pool’s exhaustion [25,26].

**Figure 1:**
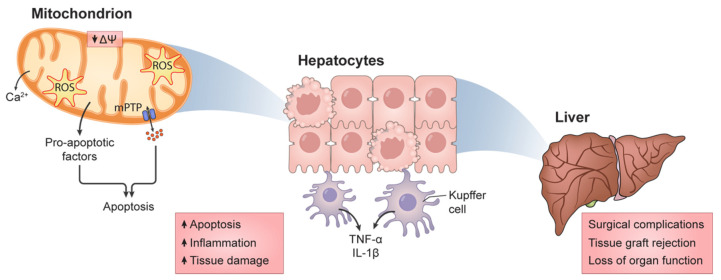
The upscaling of damage in HIRI. Severe compromise to mitochondrial function results in the exacerbated generation of reactive oxygen species (ROS), resulting in the activation of pro-apoptotic protocols such as the opening of the mitochondrial permeability transition pore (mPTP) and release of pro-apoptotic factors, such as ionic calcium (Ca^2+^). While mitochondrial Ca^2+^ release is markedly lower when compared with other Ca^2+^ sources that could lead to elevated levels (for example, the endoplasmic reticulum or from extracellular sources), the damage to mitochondrial function and integrity will undoubtedly lead to the release of Ca^2+^ and other pro-apoptotic factors. Furthermore, these sources could initiate mitochondrial dysfunction, rather than mitochondrial injury per se. Regardless, given enough replication of these phenomena, cellular survival is at risk, which in turn is a marker for further damage, due to the activation of sterile inflammatory procedures. All these processes, if left unchecked, might result in tissue loss and, eventually, organ failure.

Paradoxically, hypoxic conditions appear to create a prime environment for ROS generation, since not all of the oxygen supply is removed, but the shift towards an anaerobic metabolism is a driver for ROS generation, particularly hydrogen peroxide [27]. Figure 1 illustrates this escalation of injury, where damage to mitochondrial function and integrity escalates to tissue damage and, eventually, to organ failure.

One way by which the cellular mitochondrial population responds and adapts to fluctuating metabolic and biophysics conditions is through the modulation of mitochondrial dynamics, i.e., through alterations in mitochondrial fusion, fission, degradation and biogenesis events. These alterations result in the modification of the mitochondrial bioenergetic capacity, not only through elevated OXPHOS elements’ production, but also by more macro alterations, such as reticulation and volume [28,29]. In typical conditions, the mitochondrial network within a cell is found to be highly regulated to serve the cell’s needs (for example, mitochondria are typically found in great numbers around the nucleus). However, if an elevated energetic output is necessary, mitochondria usually undertake fission and biogenesis protocols, to elevate organelles’ numbers. In tandem, the fission process also allows for the isolation and removal, by intracellular degradation, of incompetent or damaged mitochondrial units or elements, in order to boost energetic production by the mitochondrial unit [30]. Thus, mitochondrial dynamics (i.e., the fluid shape, size and numbers) are paramount to a proper and efficient response to the ever-shifting metabolic environment and to reply to the sometimes quite different cellular necessities.

Of course, all metabolic effects deeply involve mitochondria, whereby virtually all of the cell’s ATP needs are produced, given that oxygen is available. Concomitantly, the low O_2_ pressure in ischemia all but impedes ATP generation by OXPHOS, which has immediate effects on various cellular processes, such as ion balance which, in turn, help the loss of mitochondrial membrane potential, the driving force for ATP generation in aerobiosis [15,31]. This in turn leads to the induction of what is known as the permeability transition (mPT), where smaller than 1.6 kDa (in high-conductance state) or up to 0.3 kDa (in low-conductance state) solutes can freely cross the mitochondrial membranes, including various pro-apoptotic factors [32,33], in the apparent formation of unspecific pores of dubious, possibly fluid, composition [34].

As expected, low O_2_ pressure results in a decreased mitochondrial biogenesis, as hypoxia-sensitive elements such as hypoxia-inducible factors are negative regulators of the peroxisome proliferator activated receptor gamma co-activator 1 alpha (PGC-1α), the master regulator of mitochondrial biogenesis [35]. In a similar fashion, the other dynamism fluxes are also perturbed. At the start of reperfusion, there is a drive for increased fission, due to elevated levels of dynamin-related protein 1 (Drp1) acting in harmony with other pro-apoptotic proteins such as Bax or Bak [36], a mechanism that appears to be heavily related to altered calcium metabolism (Ca^2+^ flux perturbation are discussed in more detail below). Conversely, Mitofusin protein 1 (Mfn1) and the optic atrophy protein 1 (Opa1), which are involved in the mitochondrial fusion processes, are downregulated by oxidative stress in ischemic events [37,38]. As for mitophagy, it is now ubiquitously accepted that this process is severely limited in HIRI, and that addressing it yields protection against pathogenicity [36,39,40].

As previously mentioned, one of the most affected ionic balances is the one pertaining to calcium (Ca^2+^), a ubiquitous and powerful secondary messenger as well as a cofactor for various enzymes (including some involved in cellular processes of self-degradation). As expected, given these roles, its intracellular levels are highly regulated [41]. Along with the endoplasmic reticulum, mitochondria are the main site of Ca^2+^ storage for a quick and localized release upon specific signaling processes, typically by using the mitochondrial membrane potential (ΔΨ) as a token for Ca^2+^ accumulation against the gradient [42]. In ischemia, cytosolic Ca^2+^ levels increase (partly due to the diminished ΔΨ, as mentioned above), resulting in the activation of Ca^2+^-sensitive enzymes (ex: calpains, calmodulin, protein kinase C, amongst many others), elevated oxidative stress and other deleterious events that typically result in cellular death by apoptosis [43,44].

Cellular death usually leads to the release, to the extracellular milieu, of pro-inflammatory factors that induce an immune response and overall localized aggravation of damage. In the liver, at the start (i.e., during hypoxia), Kupffer cells induce zonal injury via a combination of elevated ROS generation (generated primarily by white blood cells that are chemoattracted to the site such as polymorphonuclear neutrophils [45]) and release of pro-inflammatory cytokines such as tumor necrosis factor-alpha (TNF-α) and interleukin-1beta (IL-1β). These, in turn, lead to the attraction, migration, adhesion and chemotaxis of neutrophils, exacerbating the immune activity in the area [46,47]. However, this process further escalates, particularly after blood-flow restoration (reperfusion), partly due to the established organelle injury suffered, but also caused by the already undergoing cellular injury processes [48]. Of course, if mitochondrial function is compromised by these processes, a further escalation of injury is sure to ensue, and that is why the conservation of mitochondrial capacity and activity might tip the scale towards the survival of the cell against HIRI.

As an example of the responsibility of mitochondria in this survival is their aforementioned role in Ca^2+^ handling. As mentioned, mitochondria are not the only reservoir for Ca^2+^ storage, but simultaneously are one of the most important intracellular calcium storage locations, due to an exchange with membrane potential [49]. As such, the quick and efficient removal of Ca^2+^ from the cytosolic milieu is literally vital for the cell and, as such, mitochondria in prime conditions are mandatory for this effect, not just because of the membrane potential swap, but also because of the effects of Ca^2+^ within mitochondria. In fact, this ion’s presence in the organelle’s matrix is a major event in OXPHOS and ATP generation, since excess Ca^2+^ can oftentimes lead to mitochondrial rupture and the release of pro-apoptotic signals [49]. As such, mitochondria are a paramount player in intracellular Ca^2+^ metabolism, which is particularly relevant in non-excitable tissues that have poor endoplasmic reticulum (ER) Ca^2+^ clearance capacity and, since mitochondrial Ca^2+^ handling appears to be crucial not in homeostasis, but in situations of stress such as HIRI [42] further implicates mitochondrial function conservation as paramount for the successful recovery from HIRI. Unsurprisingly, mitochondria can be found in high numbers near Ca^2+^ release points, as to better control local ionic concentration, guaranteeing a localized Ca^2+^ effect, rather than a full cellular response, which is typically associated with events of apoptosis [49,50].

Finally, it has also been shown that mitochondria play a role in immune responses, being a central element in the metabolic transition of immune cells in proliferation as well as in inflammatory signaling [51]. Since the inflammatory process typically requires sustained oxidative stress, it is unsurprising to consider that mitochondria are considered part of the process of inflammation. In fact, their role in mediating and, in some cases, initiating the inflammatory process, was completely unknown until very recently. Recent works have shown that mitochondria actively release some of its DNA molecules (mtDNA), which are powerful pro-inflammatory elements [52,53,54]. A reason for the apparent recognition of mtDNA as an antigen might rely on the mitochondrial bacterial origin, for mtDNA is markedly over methylated when compared with nuclear DNA [55]. Furthermore, unlike the commonly depicted bacterial-like circular DNA molecule, mtDNA is in fact tightly packed with proteins, one of which is the mitochondrial transcription factor A (TFAM), which various studies have identified as a potent immunostimulator [56,57]. Curiously, there also appears to be a role for oxidative stress in this matter, as only mtDNA with oxidized bases was capable of eliciting an immune response [58]. There are many other ways by which mtDNA can be a pro-inflammatory molecule, and this topic has been excellently revised in previous research [52,55].

NF-kB, a known pro-inflammatory cytokine, has also been shown to be activated by the loss of mitochondrial membrane integrity [52]. The NOD-, LRR-, or pyrin domain-containing protein 3 (NLRP3) inflammasome proteins are ROS-dependently localized to mitochondria [59,60]. In tandem, mitochondria also have several molecules recognized by the NLRP3 inflammasome, such as cardolipin or even the mitochondrial DNA [60,61,62]. Mitochondria further enhance this inflammasome’s activity by the release of various activators (such as, for instance, cytochrome *c* or Smac), causing in turn the release of proinflammatory cytokines IL-1ß and IL-18 [60]. These pathways are apparently carefully preserved in various species, since they are fundamental following bacterial or viral infections [61,63]. However, this should be finely balanced, since these processes are also linked to highly inflammatory processes in pathologic conditions, such as HIRI [64]. There are also numerous evidences of mitochondrial roles in cancer and neurodegeneratory immunological responses (which have been extensively reviewed before [65]).

In summary, since mitochondria have a vital role in a panoply of cellular activities, it is easy to understand how mitochondrial dysfunction is intimately associated with pathological processes. As such, maintaining or restoring mitochondrial homeostasis is a widely studied and desired goal for countering various diseases, such as HIRI.

## 2. Surgical Approaches in HIRI Mitochondrial Function Compromise

In order to achieve the preservation of mitochondrial function, various surgical practices have been tested and utilized in a clinical setting for over a decade. They mostly rely on a form of damage priming, i.e., the introduction of small bouts of damage through mild, short, controlled events of removal and restoration of blood flow, in a manner that is now recognized as hormetic [30], a concept and phenomenon discussed further down. We discuss the most common ones, explaining their similarities and differences, and how they relate to mitochondrial function preservation.

### 2.1. Ischemic Preconditioning

Ischemic preconditioning (IPC) is a strategy that has shown promising results in the bench. To achieve hepatic IPC, a clamp or other means of blood flow restriction is used for a small period of time (usually 10–15 min) after which flow is restored by restrictor removal. After a proportional amount of time has passed, the procedure that will generate HIRI is then initiated [66]. While no definitive answers exist as to why IPC is a protective strategy against HIRI, some elements have been confirmed to be crucial, such as ATP levels and, thus, mitochondrial function integrity [67,68,69,70]. In tandem with preserved mitochondrial function and integrity, elevated mitophagy [71,72] and prevention of apoptosis are also key events [73,74]. In fact, not just mitophagy is important, but apparently also other cellular components’ replacement is a part of the process [75,76]. However, some criticism about the efficiency of this approach exists, because some meta-analyses have concluded that IPC is not always a guarantee of improved HIRI, since there is simply too much heterogeneity in the human population or, more clearly, in the patients studied [39,69].

It is impossible though to discuss IPC without the introduction of the concept of hormesis and, more specifically, mitohormesis. While previous work has discussed in great detail the intricacies of mitohormesis [30], it should suffice, for this works’ purpose, to acknowledge that the mitohormetic process is one where a small injurious event leads to the fairly limited but definitely present injury in mitochondria, in particular in subsets or even subsections of the organelles. These are quickly identified and removed through various mitodynamic processes such as fission and mitophagy, and are replaced by newer, more competent units, thus contributing to a more resilient cell. This is far from a novel concept or even observation, as various reports on the matter have surfaced for at least two decades, involving both intracellular events [77,78] or even the liver’s immune system [79], which is apparently also a critical participant.

### 2.2. Intermittent Clamping

A similar surgical procedure to IPC is known as intermittent clamping (IC). The main difference is the number of occlusion-flow restoration cycles and the timing of performance; whereby IPC is by default just one cycle before the HIRI event, IC consists of various cycles of flow occlusion and restoration, and these are not limited to just the pre-HIRI event. In fact, IC can be spread, even during the HIRI event [66]. In terms of hepatic HIRI, IC has been shown to be superior [80], inferior [81] and even similar to IPC [67]. These differences might fall prey to various causes, of which differences in intervention time is but one of them (different populations intervened, different medical backgrounds, etc.). However, surgery timings are possibly of the most important, for the authors of various studies have concluded that, for shorter interventions, IPC is superior as it improves transaminase levels and surgical complications but, for longer time frames, they are virtually the same procedure [60,63], for which mitochondrial function preservation and cellular ATP levels’ maintenance are imperative [82].

A variation of this procedure is ischemic post-conditioning (IPostC), where the bouts of flow restriction/restoration are performed after the main ischemic event, so it acts more like an IC but between the phases of ischemia and reperfusion [83,84]. This method results in virtually the same results as pre-HIRI IC, albeit the lack of time-consuming flow restriction/restoration events to initiate the surgical procedure might be advantageous in certain settings where there is a rush to initiate the surgical event, such as, for instance, transplantation [85,86].

### 2.3. Remote Ischemic Preconditioning

Another surgical procedure to tackle HIRI is known as remote IPC (RIPC). This is a rather bizarre phenomenon whereby events of ischemia/reperfusion localized in distant organs results in improved liver resistance to HIRI. While much is yet to be understood about this phenomenon, it is undoubtedly a reality, as many works have reported quasi-unbelievable responses of the liver (and other organs as well) to remote IPC events. While the release of protective elements (what those are is still a hotly debated topic) is probably involved and can flow in the circulation, protecting distant tissues, mitochondrial-function protection in the remote tissue/organ is apparently a necessity for RIPC in the liver [87,88,89,90,91].

### 2.4. Machine Perfusion

A technique that has attracted much attention due to its potential for use in a particular setting (transplantation of less-than-optimal tissue samples) is hypothermic machine perfusion, HMP [92]. This method’s intents are to allow for these otherwise rejected organ donations to be reconditioned and to achieve an extension of the preservation time window [86]. In this technique, the organ to be transplanted is perfused in a similar solution to the one used in normal, static cold (4 °C) storage, but with a faster flow. However, for obvious reasons, function testing is not possible, since the idea is to aggressively lower the organ’s temperature. A key modification to this protocol is to oxygenize the perfusion solution, which appears to prevent HIRI in these organs, since ROS generation and inflammation are reduced, while the ATP-generation capacity of mitochondria is salvaged [93]. While normothermic (i.e., core temperature) perfusion is both the more common method and results in lower levels of HIRI, allowing for increased graft viability (up to around 19 h), HMP has the significant advantage to help expand the pool of viable donors to otherwise unusable and certain immunorejection grafts [94].

### 2.5. Mitochondrial Transplantation

Finally, although it is not a surgical technique as classically defined, the transplantation of mitochondria is a surprisingly effective approach [95,96]. In fact, in HIRI studies, mitochondrial transplantation resulted in improved serum transaminase levels, decreased inflammatory markers and other success indicators, after mitochondria were injected directly into the spleen, from where they migrated towards the injured liver [94,97]. However, there are still many questions regarding this novel approach. It is not clear how mitochondria migrate to, enter the injured cell and start working to replace the damaged ones, but further studies on the matter will certainly help explain this matter.

## 3. Pharmacological Intervention for HIRI

It is well established that the mechanisms involved in HIRI are of a multifactorial nature, which entails complex signaling pathways. The modulation of protective/deleterious pathways using pharmacological interventions represents an attractive approach in this context, since it is theoretically cheaper and safer than surgical approaches.

Pharmacological preconditioning relies on the administration of specific drugs mimicking the protective mechanisms and biologic effects of IPC. Although the exact mechanism is still not fully understood, it seems that the protection can be achieved, at least in part, by the modulation of well characterized pathways, namely the Reperfusion Injury Salvage Kinase (RISK), the Survivor Activating Factor Enhancement (SAFE), the cyclic guanosine 3′,5′-monophosphate/ Protein Kinase G (cGMP/PKG) as well as a combination of others, including inflammatory, metabolic and mitochondrial factors (for an excellent revision on this matter, please consult [98]).

### 3.1. Mitochondrial Targeting for HIRI

#### 3.1.1. Natural Compounds

The vast majority of the pharmacological interventions involve the administration of natural (i.e., melatonin [99]), mimetic (i.e., *N*-acetylcysteine, NAC [100]), or metabolism shifting agents (i.e., trimetazidine [101] or indirubin-3′-oxime [102]). Several studies have shown the potential role of melatonin in reducing IRI. Zhang and colleagues reported that melatonin diminished myocardial IRI through the improvement of mitochondrial fusion and mitophagy, as well as the activation of AMPK-OPA1 signaling pathways [103]. Melatonin was also found to decrease IRI via the upregulation of the mitochondrial sirtuin 3 and a subsequent reduction in oxidative stress and apoptosis [104]. In addition, melatonin conditioning also alleviated hepatic IRI via the suppression of the induction of mPT [105].

Based on the crucial role of ROS in HIRI pathophysiology, pharmacological interventions aiming to neutralize or modulate its production using antioxidants were one of the first pharmacological approaches attempted. NAC is a precursor of the synthesis of glutathione, the main endogenous ROS scavenger involved in the cellular protection against oxidative stress, as well as directly scavenging pro-oxidant agents with unpaired electrons [100]. Numerous studies have reported the hepatoprotective effects of NAC administration prior to HIRI, especially via the significant reduction of transaminase release [106] and decreased oxidative stress, resulting in diminished apoptosis and autophagy [107,108].

Trimetazidine (TMZ) is a piperazine derivative that has been used as an anti-ischemic agent. The prevention of ROS production, mitochondrial damage (such as protein and phospholipid oxidation, to name a few) and decreased ATP levels are thought to be important targets by which TMZ exerts its cytoprotective effect [109], for while TMZ is not an antioxidant per se, its action in metabolic modulation results in elevated antioxidant enzyme activity [110]. The presence of TMZ confers a reduction in hepatic injury and an improved fatty liver functionality after ischemia/reperfusion, through the activation of AMPK and subsequent increase of nitric oxide levels [111].

The pharmacological preconditioning with indirubin-3′-oxime was demonstrated to protect the liver against HIRI by preserving mitochondrial function and hepatic energetic balance [112]. This compound inhibits GSK-3β and, consequently, prevents cyclophilin D phosphorylation by GSK-3β. Since cyclophilin D serine residues’ phosphorylation leads to mPT induction [112,113], its inhibition modulates the susceptibility to mPT induction, thus preserving mitochondrial function following HIRI.

#### 3.1.2. Synthetic and Directed Agents

##### OXPHOS Elements Manipulation

Succinate accumulation has been heavily implied in HIRI [114]. By using succinate dehydrogenase (OXPHOS Complex II) inhibitors such as malonate, succinate accumulation is prevented and thus ischemic injury is reduced [114]. Similarly, Complex I inhibitors have been investigated. It is true that a wide panoply of mitochondrial-beneficial agents have Complex I inhibitory capacity (ex: Metformin, Berberine, to name a few) but those appear to rely on mitohormetic effects; here, other types of molecules such as MitoSNO (mitochondrial-specific S-nitrosating agent) or Amobarbital have proven protection in ischemia/reperfusion [115,116], effects that might depend on diminished pro-apoptotic factors’ release for the prevention of retrograde electron flow and thus increased ROS generation [36].

##### Mitochondrially Targeted Antioxidants

Unlike the aforementioned NAC, which has a wider range of activity than just mitochondria, other antioxidants specifically designed to target mitochondria have already been tested for the same reasons. For instance, Coenzyme Q_10_ (or ubiquinol) is not only a member of the respiratory chain but also a potent antioxidant, which has proven to be able to prevent HIRI [117,118]. MitoQ and SkQ1 are other mitochondrial-specific antioxidant molecules, modelled after the widely known tetraphenylphosphonium (TPP^+^), a molecule that freely traverses the inner mitochondrial membrane and, as such, this property was explored by various researchers to deliver antioxidant agents to the mitochondrial matrix [119,120]. Other classes of compounds, with different antioxidant roots and delivery alternatives exist and most have shown similarly effective results, such as Bendavia (a Szeto-Schiller peptide [121]), MitoGSH [122], or Euk-8 [123] although not all exactly on HIRI, but on other tissues/organs, which opens the possibility for more studies.

##### NAD^+^ Metabolism

As previously mentioned, NAD^+^ is an essential co-factor to many metabolic reactions, and in low abundance in HIRI. To further complicate matters, NAD^+^ transport across biological membranes is a very complicated procedure, and oftentimes impossible although in recent years some transporters have been identified [124]. As such, most studies have focused on more mobile NAD+ biosynthesis precursors nicotinamide riboside (NR) and nicotinamide mononucleotide (NMN). Their use boosts the cell’s NAD^+^ pool, a feature already explored in various works [125,126].

##### PPAR Agonists

Another strategy that is not immediately intuitive is the use of agonists of the nuclear receptor family of peroxisome proliferator-activated receptor. For instance, fibrates that directly activate PPARα, a commonly used antidiabetic class of drugs, have been shown to attenuate HIRI [127]. Even more striking is the fact that the PPARγ agonist pioglitazone has very interesting anti HIRI potential [128]. It is far from clear how this class of molecules achieve these results, but so far, a purely antioxidant effect has been shown [36].

##### Mitochondrial Dynamics Modulators

As previously discussed, mitochondrial dynamics play a significant role in HIRI. As such, it was only logical to target these processes to discover potential clinical avenues. In fact, just by boosting mitochondrial biogenesis it was possible to prevent damage associated with HIRI. Various works on this matter used different strategies to achieve this (examples are by using the AMP-activated kinase, AMPK, agonist AICAR [129], or by using the recently identified hormone irisin [130], or even using natural compounds such as the flavonoid nobiletin [131]). Regardless of the strategy, the result was always the increased PGC-1α activity, thus increasing mitochondrial numbers and preventing the drop of ATP in HIRI.

If by increasing mitochondrial levels these works demonstrated a prevention of HIRI, the same can also be said when blocking mitochondrial fission. By targeting the dynamin-related protein 1 (Drp1) translocation to mitochondria with Mdivi-1, a quinazolinone that is commonly used to inhibit fission, Yu and colleagues were able to alleviate mitochondrial fragmentation and reduce apoptosis [132]. This reduction in mitochondrial fission was also found and associated in a previously mentioned work by Bi and collaborators [130]. Since mitochondrial fission is a hallmark of post-ischemic injury, the prevention of excessive fission appears to be a fairly efficient strategy to reduce HIRI.

However, not all mitochondria are unscathed in HIRI, and their removal and replacement by non-injured ones could be essential for surviving this phenomenon. The mechanistic target of rapamycin (mTOR) is a rapamycin-sensitive central regulator of the metabolism, and its activity has been negatively associated with mitophagy [133]. As such, it is unsurprising that rapamycin has been reported to boost mitophagy and substantially prevent HIRI [134]. Similarly, the silencing of the *park7* gene that codes for the protein deglycase DJ-1 also protects the liver against HIRI by boosting mitophagy [135]. Comparatively, the augmenter of liver regeneration (ALR) is an anti-apoptotic protein primarily found in mitochondria, which has been found to boost mitophagy by driving up the expression of mitofusin 2, protecting against HIRI [71]. Finally, boosting mitophagy with natural compounds is also analogously efficient, since the polyphenol pterostilbene has also been found to protect the liver against HIRI by upregulating the Parkin-PINK1 pathway of mitophagy [136].

##### mPT Inhibiton

Even though taking different routes, many of the pathways involved in HIRI culminate in critical events, and the most well recognized among them is the mPT pore opening. Aside from the already mentioned natural compounds whose activity results in mPT inhibition, several other agents that achieve this same goal were tested. In fact, the widely known mPT pore opening inhibitor Cyclosporin A has been known to protect the liver against HIRI for decades [137,138]. Other molecules, similar to Cyclosporin A in function but without some of its more undesirable side effects (such as immunosuppression) have also been tested, such as Sanglifehrin or NIM811 [139,140].

These are just a few examples of intense research on the field of mitochondrial-function manipulation to combat HIRI. However, no definitive pharmacological agent has proven to be a widespread, safe and effective clinical agent. Thus, the development of new, more efficient and safe pharmacological strategies aiming to improve mitochondrial function might be a viable approach to mitigate the hepatic damage underlying HIRI.

### 3.2. Mitochondrial Aldehyde Dehydrogenase 2 as a Therapeutical Target

Aldehyde dehydrogenase 2 (ALDH2) is a mitochondrial enzyme, mostly expressed in the liver, where it plays a pivotal role in ethanol metabolism [141]. In addition, ALDH2 is also involved in the clearance of toxic aldehydes originated from the lipoperoxidation of mitochondrial and plasma membranes, mainly 4-hydroxynonenal (4-HNE) and malondialdehyde (MDA), under oxidative stress conditions.

Furthermore, Alda-1 is a well-known ALDH2 activator, and several reports have demonstrated a significant improvement against IRI in the presence of Alda-1 in various types of organs, including heart, brain, lung, kidney and intestine [142,143,144].

Recently, Li and colleagues reported the protective effect of Alda-1 against HIRI in mice [145]. The authors suggested that the protective mechanism was related to the clearance of reactive aldehydes (decreased accumulation of 4HNE and MDA) and autophagy enhancement by AMPK activation. These results suggest that Alda-1 pretreatment could increase ALDH2 activity, which in turn scavenges reactive aldehydes.

In accordance, Alda-1 pretreatment protected the liver in a rat model of HIRI, resulting in decreased hepatic enzyme release, oxidative stress and inflammation, through autophagy enhancement which might be dependent on the AKT/mTOR and AMPK signaling pathways [146]. Moreover, reduction in the liver mitochondrial damage and attenuation of hepatocyte apoptosis were also observed.

These results demonstrate that ALDH2 might be a possible target for pharmacological strategies in a context of HIRI in clinical practice.

### 3.3. Polyethylene Glycol: A New Promising Approach

Polyethylene glycols (PEGs) have shown multiple benefits in cell and organ preservation, including antioxidant capacity, edema prevention and plasma-membrane stabilization [147]. Besides being widely used as oncotic agents in preservation and rinse solution [148], a sizeable body of literature have been demonstrating the beneficial effects of PEGs against IRI by promoting the protection of mitochondria and cytoskeleton [98]. In addition, both in vitro and in vivo studies demonstrated that the high molecular weight PEGs can reduce cytokine production and neutrophil activation [149,150].

Furthermore, PEG35 has been linked to increased levels of ALDH2 and improved mitochondrial machinery, diminishing the ischemic injury [151,152]. Recently, Bardallo and colleagues demonstrated an improved hepatic protection against an ischemic insult by the reduction of oxidative stress through ALDH2 upregulation and the consequent promotion of cytoprotective autophagy in a PEG35-dependent manner [153].

PEG preconditioning was also reported to protect cardiomyocytes from hypoxia and reoxygenation-induced cell death. The protective effect is suggested to be linked to PEG’s capacity to decrease ROS production and lipoperoxidation, which in turn leads to membrane stabilization and consequent maintenance of cell integrity and inhibition of apoptotic cell death. PEG is capable of sealing and progressively eliminate membrane disruptions [154]. Still, some PEG molecules can pass through the membrane openings and interact with the mitochondrial membrane, preventing the induction of mPT [155]. Consequently, there is a reduction in mitochondrial swelling, which allows for the maintenance of mitochondrial membrane potential and inhibition of cytochrome *c* (Cyt. *C*) release and subsequent apoptosis induction. Furthermore, PEG postconditioning improved myocardial protection in an in vivo model of rat hearts subjected to 1 hour of artery occlusion followed by either 48 hours or 4 weeks reperfusion [156]. The mortality within the first 24 hours had significantly better outcome in the presence of PEG, as 40% of the animals died in non-PEG group against only 10% in the PEG-treated rats.

In the liver, PEG35 preconditioning ameliorated hepatic injury and protected mitochondria in a rat model of cold ischemia followed by warm reperfusion (4 and 37 °C, respectively) [157]. The protective effect was associated with the stimulation of the pro-survival pathway via AKT phosphorylation as well as the activation of two important cytoprotective mediators, e-NOS and AMPK. Bejaoui and colleagues have also shown PEG35 hepatoprotective effects against warm HIRI. Additionally, PEG35 preconditioning efficiently decreased transaminase levels and maintained hepatocyte morphology as well as preserved mitochondrial membrane potential. The activation of the pro-survival kinase Akt and the cytoprotective factor AMPK and the inhibition of apoptosis were the protective mechanisms correlated with PEG35 protection in this study [158].

In summary, PEG35 might be considered a suitable pharmacological agent against HIRI in a clinical setting. In fact, PEG is already in use in various formulations of hepatic preservation solutions and, more specifically, PEG35 is also showing fast-tracking potential [159].

## 4. HIRI and Transplantation

The advances in surgical techniques and immunosuppressive drugs have established liver transplantation as a standard mainstay curative therapy in end-stage liver disease. Organ transplantation is a complex procedure associated with multifactorial reasons which may end up in graft failure, such as donor factors (i.e., age, steatosis, or donation after death), organ retrieval and preservation (cold and warm ischemic times), and the transplantation procedure itself, including surgeon expertise as well as possible surgical complications [160].

The gap between the number of patients waiting for liver transplantation and the number of available organs has drastically increased (due to increased demand), leading to an urgent need for the expansion of the donor pool to match the growing need for organs. In this sense, the possible use of suboptimal or marginal organs as a viable approach to augment the number of available organs for transplantation has been highly encouraged. However, this expanded criteria donor (ECD) organs are well known for presenting a higher vulnerability against HIRI and their use increases primary nonfunction and compromises graft outcome after transplantation [161]. Therefore, the challenge in the transplantation community is look for new therapeutic strategies to minimize factors that overall render the organ non-functional and at the same time, increase the use of marginal livers for transplantation instead of discarding them.

The use of cold preservation solutions is the main technique in organ transplantation to maintain the morphological and functional integrity of the graft. It has the purpose of reducing, as much as possible, factors that compromise graft quality, especially the ones implicated in HIRI and the complications deriving from it. The composition of a preservation solution will dictate the quality and duration of graft preservation, through the prevention of energy depletion, acidosis, edema and oxidation, among others [162,163,164,165].

Throughout the transplantation process, the liver faces events of warm, cold and rewarming ischemia which lead to organ damage. Briefly, when the organ is retrieved from the donor and stored within the preservation solution under hypothermic (4 °C) conditions, it undergoes a period of cold ischemia. Once removed from cold storage, the graft is exposed to warm (22–25 °C) ischemia before the beginning of reperfusion into the recipient. Consequently, the cumulative injury resulting from the combined action of ischemia and cold preservation must be minimized in order to achieve the maximal possible recovery of the graft’s function after liver transplantation.

Currently, there are two main strategies for organ preservation, namely static and dynamic. Static Cold Storage (SCS) consists of organ preservation at low temperatures (0–4 °C) to reduce metabolic activities and consequently cellular damage [166]. Hypothermia is well known to significantly decrease cellular metabolic rates and, on average, oxygen and glucose requirements decrease up to 8% for every degree of temperature lowered [167]. Consequently, cold storage intends to delay the depletion of ATP levels and slow down the deleterious processes associated with ischemia. In SCS, the organ is perfused with a cold solution and is afterwards statically stored in a container filled with the cold preservation solution while waiting for transplantation. Regarding dynamic perfusion, the retrieved organ is placed in a chamber, in which it is continuously perfused with either an oxygenated or non-oxygenated solution using a machine perfusion (MP) pump, as previously mentioned. The continuous perfusion allows for a better distribution of the preservation solution throughout the graft, as well as blood washout, continuous delivery of oxygen and nutrients and toxic metabolites clearance and, as a result, a better outcome [168,169]. In addition, this technique also allows for a real-time monitorization of the functional and biochemical performance of the graft as well as the possibility of the application of a pharmacologic agent [170]. MP can be performed under different temperatures, including Hypothermic Machine Perfusion (HMP), Normothermic Machine Perfusion (NMP), Subnormothermic Machine Perfusion (SNMP) and Hypothermic Oxygenated Perfusion (HOPE), as previously discussed [171].

### 4.1. Static Cold Preservation

The maintenance of organ viability during cold storage is of utmost importance to a successful outcome after liver transplantation. Preservation solutions were initially developed decades ago to minimize graft injury during cold storage. The first preservation solution was developed by Collins and co-workers in 1969 and intended to mimic the intracellular composition and protect the intracellular spaces during the onset of ischemia [172]. A few years later, the University of Wisconsin (UW) solution was developed and has since been considered the gold standard for hepatic preservation solutions. However, its high potassium concentration induces cellular depolarization as well as vasoconstriction, which impairs the organ perfusion during washout and reperfusion [173]. In addition, one of its components, the oncotic agent hydroxyethyl starch (HES), has been associated with red blood aggregation, macrophage invasion and tubular damage [174,175].

The replacement of HES for another oncotic agent (PEG20 kDa) in the UW solution was reported to improve rabbit-heart performance after 24 h preservation [176]. In addition, liver grafts stored in UW solution and then rinsed with a solution containing PEG35 showed reduced hepatic injury and improved liver function after reperfusion [148]. The better outcome was associated with the prevention of oxidative stress, mitochondrial damage and liver autophagy.

The beneficial effects of PEGs have been known for decades. Back in the 1970s, Daniel and Wakerley demonstrated increased cellular viability using PEG 20 kDa during the cold preservation of renal pig cells [177], whereas lower molecular-weight PEG (6 kDa) protected myocardium from cellular edema and membrane damage during preservation [178]. Since then, several studies have demonstrated the protective role of different molecular weight PEG during cold preservation using different animal models and the satisfying results obtained led the use of PEG to a clinical level. In France, a solution similar to UW solution was developed in the beginning of the century: the Institute Georges Lopez (IGL)-1 solution. Contrary to UW solution, IGL-1 is characterized by high sodium and low potassium concentrations and the presence of PEG35 as a colloid [142], and it has been considered as a suitable alternative to UW solution by the European Liver Transplant Registry [179] due to and its efficiency in the preservation of abdominal organ [180,181,182].

Liver grafts preserved in IGL-1 solution have been found to possess increased ALDH2 expression and activity, which in turn were associated with reduced mitochondrial damage and ATP breakdown prevention [152]. In accordance, the presence of PEG35 in the IGL-1 solution was reported to be a crucial agent in mitochondria preservation and in the reduction of hepatic injury, when compared liver graft preservation using IGL-1 and IGL-0 (same composition than IGL-1, but without the presence of PEG35) solutions [159]. Accumulating evidence demonstrates the efficacy of PEGs in upregulating cell-survival pathways in distinct tissues, resulting in mitochondria and cellular membrane protection and the prevention of ROS production and cell swelling [149,156,157,183,184]. Despite IGL-1′s beneficial effects, the underlying protective mechanisms are rather complex and not completely understood. They may include cytoprotective mechanisms exerted, at least in part, by the upregulation of ALDH2 and subsequent activation of AMPK (cytoprotective factor), Nitric oxide (NO) generation (vasodilator agent), as well as the aforementioned mitochondrial protection, among others [152,185].

Recently, a new IGL solution (IGL-2) was designed, and the main differences to the previous IGL solution are a higher concentration of PEG35 and glutathione. In order to understand the direct role of PEG35, Bardallo and colleagues evaluated liver graft preservation using 3 IGL solutions, IGL-0, IGL-1 and IGL2, containing 0 g/L, 1 g/L and 5 g/L of PEG35, respectively. The authors found that the presence of PEG35 seemed to be a major factor in preventing hepatic injury in a dose-dependent manner. In addition, a superior antioxidant capacity of IGL-2 solution against ischemic insult during liver graft preservation was also observed, most likely through ALDH2 upregulation and the subsequent promotion of cytoprotective autophagy [153]. The main characteristics and components of the discussed and more common preservation solutions are detailed in Table 1.

### 4.2. Dynamic PreservationRe

As previously mentioned, the need for organs is constantly increasing and thus, the use of novel techniques for optimizing suboptimal graft preservation is of utmost importance. Machine perfusion devices are not a novel technology, but were scarcely used for organ preservation, mainly due to logistical issues. Nowadays, with the advancement in innovation and technology, their design is more portable and efficient, and consequently, provides a more promising therapeutic strategy for graft preservation.

As mentioned above, oxygen and mitochondria play an important role during HIRI. Hepatic perfusion in HOPE benefits in great extent of the oxygenation of the perfusate, which is responsible for maintaining the integrity and function of the mitochondrial population [186]. In this review, we will only address the dynamic preservation under HOPE conditions. As for other modes of dynamic organ preservation (HMP, NMP, SNMP), an excellent work has been previously published [187].

MP demonstrated to be more protective than static cold storage in a comparison study of DCD (donation after circulatory death) liver grafts [188]. HOPE combines the benefits of cold preservation conditions with active oxygenation of the perfusate, enabling graft’s mitochondria the capacity to produce and restore ATP to levels similar to before reperfusion, which significantly increase within the first hour of perfusion [189]. Moreover, cold oxygenation also reduces mitochondrial ROS release, triggering less oxidative damage in mitochondria in early reperfusion [190]. Furthermore, the accumulation of some metabolites such as succinate, during the ischemic period have been reported to provoke mitochondrial dysfunction on several tissues [191]. Hence, the removal of such metabolites by the dynamic flow observed in HOPE might be an important way to increase the odds of a proper mitochondrial function during early normothermic reperfusion.

Kron and colleagues reported the protective effects of HOPE in fatty liver grafts in rats and humans [192]. SCS, followed by 1h HOPE treatment was found to protect the liver from initial ROS generation and DAMPs release after transplantation, as well as decreased activation of inflammatory pathways. Moreover, recovery of ATP levels prior to reperfusion and reduction of cell death during reperfusion have also been reported after the HOPE period [193].

Belzer Machine Perfusion Solution (Belzer MPS) and its generics, which are a variation of the original UW solution used in the SCS, are the most commonly used perfusion solution for HOPE. However, the use of Belzer MPS in liver machine perfusion has some limitations. For instance, given its high viscosity, Belzer MPS may lead to sinusoidal shear stress, which can induce the destruction of the glycocalyx of hepatocytes [194]. Glycocalyx comprises the thin luminal sugar monolayer that protects the graft endothelia and its damage has been related to graft injury and function in clinical liver transplantation [195,196].

**Figure 2:**
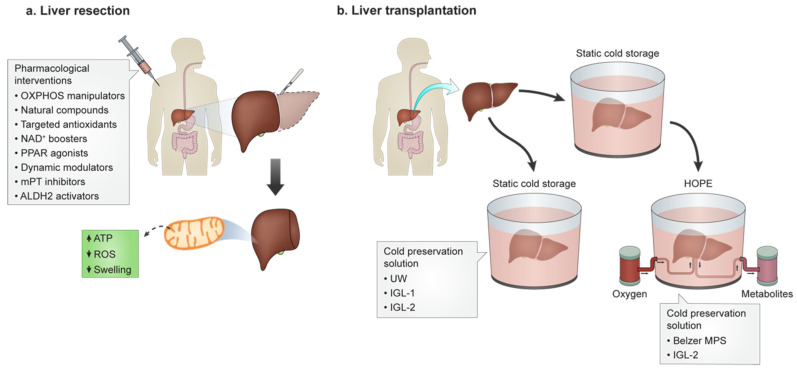
Interventions towards improved hepatic survival in liver surgery upon HIRI. (**a**) in liver surgeries where warm ischemia is required (for instance, in liver resection surgeries), preconditioning with mechanical (i.e., blood flow occlusion and restoration) or with pharmacological agents have been tested both in the bench and the bedside, with promising results. Of note is that, by directly or indirectly targeting mitochondria for function preservation, these strategies can make up for the difference in between failure or preservation and restoration of hepatic function, size and proficiency. (**b**) On the other hand, where cold ischemia is required (i.e., transplantation), new developments in preservation solutions have both increased the time window for which the organ is still in usable form, and also expanded the pool of organs that can be used, by virtue of the massively improved preservation protocols.

As previously mentioned, HOPE has a proven effect in mitochondrial protection in dynamic preservation, while PEG35 has been demonstrated to protect mitochondria in static preservation. In this sense, the new IGL-2 solution has been proposed as the perfusate for HOPE. A comparison study using Belzer MPS and the new IGL-2 solution for 1h of HOPE after SCS in a rat model, revealed a significant prevention of mitochondrial damage in the IGL-2 HOPE conditions. The authors suggested that IGL-2 could be a suitable tool in HOPE strategies, especially for rescuing vulnerable liver grafts such as the steatotic ones for transplantation [159]. These procedures are represented in Figure 2.

## 5. Concluding Remarks

It is now clear that many of the cellular and mitochondrial events in HIRI are also present in other organs/tissues subjected to a similar event. This is extremely encouraging and helpful, as it means that virtually all of the research conducted in this field of ischemia/reperfusion is highly transversal between organs, saving both time and resources [98]. From all of the explored literature, patterns of intervention begin to emerge, which have been mentioned in this work. Most strategies would thus benefit from a combination of the main goals of the works discussed here, ranging from reduced oxidative stress from the earliest possible time to the elevation of mitochondrial numbers, activity and resilience.

## Figures and Tables

**Table 1:** Static Cold Storage and Dynamic preservation solutions compositions.

Components	UW	IGL-1	IGL-2	Celsior	Belzer-MPS
K^+^ (mmol/L)	125	25	25	15	25
Na^+^ (mmol/L)	27	125	125	100	120
Mg^2+^ (mmol/L)	5	5	5	13	5
SO_4_^2−^ (mmol/L)	4	5	5	-	5
Ca^2+^ (mmol/L)	-	0.5	-	0.25	0.5
Cl^−^ (mmol/L)	-	-	-	40	-
Zn^2+^ (mmol/L)	-	-	0.091	-	-
Diphosphate (mmol/L)	25	25	25	-	25
HEPES (mmol/L)	-	-	-	-	10
Histidine (mmol/L)	-	-	30	30	-
Raffinose	-	30	-	-	-
Mannitol (mmol/L)	-	-	60	60	30
Lactobionic acid (mmol/L)	105	100	80	80	-
Dextrose (mmol/L)	-	-	-	-	10
Ribose (mmol/L)	-	-	-	-	5
Gluconate (mmol/L)	-	-	-	-	85
Hydroxyethyl starch (g/L)	50	-	-	-	50
Polyethylene glycol 35 (g/L)	-	1	5	-	-
Glutathione (mmol/L)	3	3	9	3	3
Allopurinol	-	1	-	-	-
Adenosine (mmol/L)	5	5	5	-	-
Glutamic acid (mmol/L)	-	-	-	20	-
Adenine (mmol/L)	-	-	-	-	5
NaNO_2_ (nmol/L)	-	-	50	-	-
pH	7.4	7.4	7.4	7.4	7.4
Osmolarity (mosmol/L)	320	320	320	320	320

The concentration of some components may vary among manufacturers.
